# Parallel Guidewire for Catheter Stabilization in Interventional Radiology: The Anchoring Wire Technique

**DOI:** 10.5334/jbsr.1890

**Published:** 2020-01-15

**Authors:** Ihsan Moslemi, Haytham Derbel, Mélanie Chiaradia, Fabrice Deprez, Manuel Vitellius, Hicham Kobeiter, Vania Tacher

**Affiliations:** 1 CHU Henri Mondor, FR; 2 CHU UCL Namur, BE

**Keywords:** Interventional-Vascular, Catheters, Chemoembolization, Embolization, Education

## Abstract

This technical note describes the parallel guidewire method: the anchoring technique as a strategy to ease difficult catheterization in various endovascular interventions. Sixteen patients were included in 2017 in whom this technique was used. The type of intervention, the nature of the target and anchored vessels and possible complications on the anchored vessel were reported. This study included thirteen various embolization cases and four visceral vessels angioplasties cases. The success of catheterization by using this technique was achieved in all cases, without complication on the anchored vessels.

## Introduction

Stability of a catheterization is a crucial factor for technical success in endovascular interventions. Particularly in tortuous vascular anatomy, progression of catheter and/or microcatheter over the guidewire (co-axial technique) may be difficult or impossible, due to retrograde bascule (or a kickback) of the guidewire, the catheter and/or the microcatheter outside the target vessel. Parallel guidewire stabilization techniques have been described in specific interventions for navigation in tortuous cervicoencephalic vessels [1], in complex anatomy of pulmonary artery [2] and in femoral and peroneal arteries [3]. This technique is well established in interventional cardiology to treat chronic total occlusion of coronary artery [4,5], with the use of double lumen catheters. The latter technique involves placing a second support wire in a proximal side branch to increase the guiding catheter stabilization for revascularization.

The parallel guidewire stabilization technique keeps access during complex or difficult cases and enables stable position of the introducer into a target vessel.

The aim of this technical note was to describe the anchoring technique and report cases in whom the use of this technique was needed in our experience in a year.

## Technique

In 2017, 16 patients underwent anchoring technique for endovascular intervention in our department. Patients’ characteristics and interventional parameters are reported in Table 1.

**Table 1:** Summary of cases needing the anchoring wire technique.

Patient	Age (years)	Type ofintervention	Anchored vessel	Target vessel	Procedure time (minutes)	Fluoroscopy time (minutes)	DAP (Gy.cm^2^)

1	54	TACE	SMA	Right hepatic artery	95	45	100
2	62	TACE	SMA	Pancreatic arcad	165	48	245
3	80	TACE	Hepatic artery	Left gastric artery	85	55	505
4	51	TACE	Right renal artery	Right adrenal artery	60	21	302
5	48	Digestive bleeding embolization	SMA	Jejunal branches	72	30	55
6	77	Digestive bleeding embolization	SMA	SMA branch	86	23	123
7	80	Digestive bleeding embolization	SMA	DPA	NA	NA	NA
8	49	Duodenopancreatic artery pseudo-aneurysm embolization	Splenic artery	CHA	NA	49	175
9	61	Hepatic pseudo-aneurysm embolization	Splenic artery	CHA	107	33	310
10	75	Dorsal pancreatic artery aneurysm embolization	DPA	Dorsal pancreatic artery	103	24	50
11	68	Celiac trunk aneurysm angioplasty (with stent)	Splenic artery	CHA	72	44	111
12	59	Hepatic artery pseudo-aneurysm embolization	Splenic artery	Hepatic artery	76	NA	NA
12	59	Hepatic artery of the graft angioplasty (with stent)	Splenic artery	Hepatic artery	53	NA	NA
13	70	Hepatic artery of the graft angioplasty (without stent)	Splenic artery	Hepatic artery	56	19	89
14	66	Hepatic veins angioplasty and hepatic biopsy	Inferior vena cava	Hepatic veins	56	20	123
15	52	Renal angiomyolipoma embolization	Left renal artery	Left adrenal artery	76	36	152
16	73	Bone hypervascular metastasis embolization	Right femoral artery	Right profunda femoral artery	256	49	148

CHA: common hepatic artery; DAP: dose area product; DPA: duodenopancreatic arcad; NA: not available; SMA: superior mesenteric artery; TACE: transarterial chemoembolization.

### Details of the Parallel Guidewire Anchoring Technique

After failure of standard technique with various coaxial catheters, the senior interventional radiologist (with more than five years of experience) decided to use the anchoring technique.

**Figure 1:**
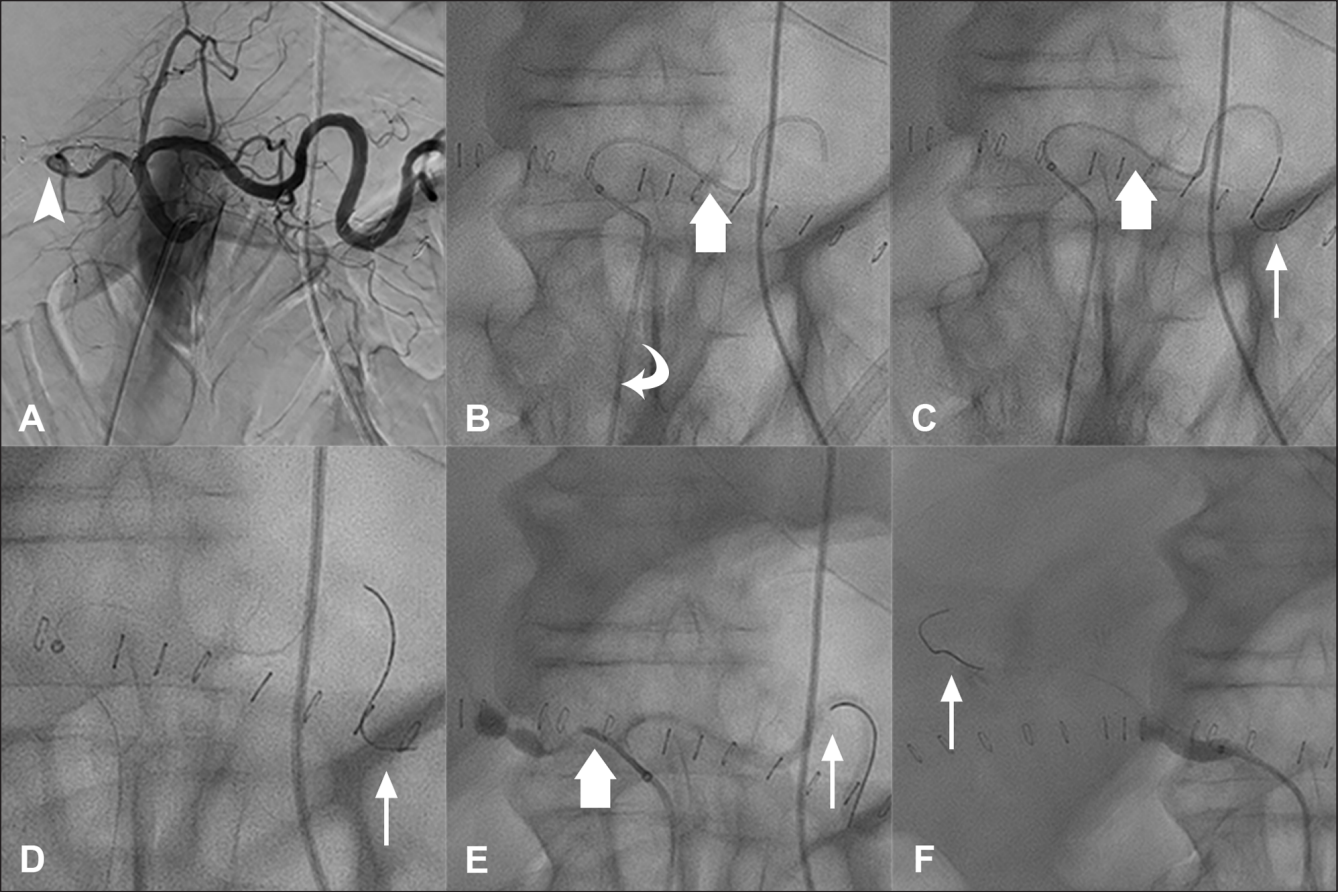
Details of the Parallel Guidewire Anchoring Technique. Angioplasty without stenting of the graft hepatic artery in a 70-year-old patient. **A.** Initial arteriography showed a graft hepatic artery stenosis (arrowhead). **B.** Catheterization was performed as distally as possible in the splenic artery with a catheter and a microcatheter (large arrow) through a long sheath (curved arrow). **C.** A stiff 0.014” guidewire (thin arrow) was introduced into the microcatheter (large arrow). **D.** The microcatheter and the catheter were completely removed. **E.** The catheter and the microcatheter (large arrow) were introduced in parallel of the stiff guidewire and used to catheterize the common hepatic artery. **F.** The stiff guidewire (thin arrow) was removed from the splenic artery to position the long sheath further into the graft hepatic artery.

The steps of the technique are listed below in an illustrative case of an intervention in the hepatic arterial tree, in a patient with an acute angle of the celiac trunk due to a median accurate ligament (Figure 1A):

1. The femoral short introducer was replaced by a long sheath.
2. The catheterization was performed as distally as possible into the splenic artery with a catheter and a microcatheter (Figure 1B).
3. A stiff 0.014” guidewire was introduced into the microcatheter to straighten the system and to enable maximum stability: the splenic artery became the “anchored” vessel (Figure 1C).
4. The catheter was pushed as far as possible over the microcatheter.
5. The long sheath was pushed into the proximal portion of the splenic artery over the catheter.
6. The microcatheter and the catheter were completely removed (Figure 1D).
7. The long sheath was pulled slowly while injecting iodinated contrast until the opacification of a targeted vessel, here, the common hepatic artery.
8. The catheter and the microcatheter were introduced in parallel of the stiff guidewire and used to catheterize the common hepatic artery (Figure 1E). The target vessel was catheterized with the catheter and the microcatheter, and the intervention was performed. In case of the need to position the long sheath further into the target vessel, the stiff guidewire was then removed (Figure 1F).

## Results and Discussion

The parallel guidewire anchoring technique was used in 17 cases in 16 patients. Sixteen interventions were performed with femoral approach and one with jugular approach. The anchoring technique was mainly used in embolization cases (n = 13) and in angioplasty cases (arterial and venous, n = 4). The anchored vessels were mainly splenic artery (n = 6) and superior mesenteric artery (n = 5), but also hepatic artery (n = 1), duodenopancreatic arcad (n = 1), renal artery (n = 2), femoral artery (n = 1) and inferior vena cava (n = 1).

Catheterization was achieved in all cases. No complication (such as dissection or thrombosis) occurred in the anchored vessel.

This technique may avoid the risk of failure, the need of another vascular access with known complications, a prolonged intervention and a long X-ray exposure. The guidewire used in parallel has to be stiff enough to enable stability for long sheath, catheter, microcatheter and guidewire placements. The use of long sheath increased stability when using this technique especially to introduce balloon, stent or embolic agent. Tension in the co-axial equipment due to acute angulation and/or tortuosity is reduced by the alignment of the afferent and the anchored vessels induced by the stiffness of the placed guidewire.

In our study, most of interventions were performed from femoral approach but this technique may be applied to others approaches, as humeral or radial approach.

Other techniques of stabilization, using different kind of devices have been described. One of the most widespread technique is the use of an angioplasty balloon, especially in interventional cardiology for chronic total occlusion treatment [6,7]. The use of the wire anchoring technique more than the balloon anchoring technique seems to be easier, faster, cheaper, and safer (regarding complication such as vessel rupture risk).

In specific embolization cases, Amplatzer Vascular Plug anchoring technique has been reported [8].

The parallel guidewire anchoring technique is, in our experience, a convenient method in cases with difficult catheter stabilization in various endovascular interventions. This technique, known to experienced interventional radiologists, has never been described in the literature in these procedures. Thus, it would benefit from being better known, particularly by less experienced interventional radiologists or those using other stabilization techniques, in order to expand their panel.
